# A Multifactorial Role for *P. falciparum* Malaria in Endemic Burkitt's Lymphoma Pathogenesis

**DOI:** 10.1371/journal.ppat.1004170

**Published:** 2014-05-29

**Authors:** Charles Torgbor, Peter Awuah, Kirk Deitsch, Parisa Kalantari, Karen A. Duca, David A. Thorley-Lawson

**Affiliations:** 1 Tufts University School of Medicine, Boston, Massachusetts, United States of America; 2 Department of Biochemistry and Biotechnology, Kwame Nkrumah University of Science and Technology (KNUST) and Kumasi Centre for Collaborative Research, Kumasi, Ghana; 3 EENT Clinic, Komfo Anokye Teaching Hospital (KATH) and PAKS Hospital, Kumasi, Ghana; 4 Department of Microbiology and Immunology, Weill Cornell Medical College, New York, New York, United States of America; 5 University of Massachusetts Medical School, Department of Medicine, Division of Immunology and Infectious Diseases, Worcester, Massachusetts, United States of America; University of Zurich, Switzerland

## Abstract

Endemic Burkitt's lymphoma (eBL) arises from the germinal center (GC). It is a common tumor of young children in tropical Africa and its occurrence is closely linked geographically with the incidence of *P. falciparum* malaria. This association was noted more than 50 years ago. Since then we have learned that eBL contains the oncogenic herpes virus Epstein-Barr virus (EBV) and a defining translocation that activates the c-myc oncogene. However the link to malaria has never been explained. Here we provide evidence for a mechanism arising in the GC to explain this association. Accumulated evidence suggests that eBL arises in the GC when deregulated expression of AID (Activation-induced cytidine deaminase) causes a c-myc translocation in a cell latently infected with Epstein-Barr virus (EBV). Here we show that *P. falciparum* targets GC B cells via multiple pathways to increase the risk of eBL. 1. It causes deregulated expression of AID, thereby increasing the risk of a c-myc translocation. 2. It increases the number of B cells transiting the GC. 3. It dramatically increases the frequency of these cells that are infected with EBV and therefore protected from c-myc induced apoptosis. We propose that these activities combine synergistically to dramatically increase the incidence of eBL in individuals infected with malaria.

## Author Summary

Endemic Burkitt's lymphoma (eBL) is a common tumor of young children in tropical Africa that is closely linked geographically with *P. falciparum* malaria. This association was noted more than 50 years ago. Since then we have learned that eBL contains the oncogenic herpes virus Epstein-Barr virus and a defining translocation that activates the c-myc oncogene. However the link to malaria has never been explained. Here we show that malaria has multiple effects that all focus on germinal center (GC) B cells that are known to be the origin of eBL. Together these effects of malaria act synergistically to dramatically increase the risk of developing eBL in individuals infected with the parasite. Clinical interventions that lessen the impact of malaria on GC B cells should dramatically decrease the incidence eBL.

## Introduction

Endemic Burkitt's lymphoma (eBL) is an extremely common tumor of young children in tropical Africa [1]. Genetic, phenotypic and transcriptional analysis suggests that it originates from germinal center (GC) cells [2], [3] although it actually grows in extrafollicular locations. It is defined by a well described chromosomal translocation between the c-myc oncogene and one of the immunoglobulin loci that results in constitutive activation of the oncogene leading to uncontrolled growth of the cell [4], [5], [6]. Recent studies indicate that this translocation may be mediated as a consequence of deregulated expression of the enzyme AID (Activation-induced cytidine deaminase) [7], [8], [9]. AID is highly expressed in GC B cells and is normally responsible for the processes of somatic hypermutation and class switch recombination of immunoglobulin genes as they undergo affinity maturation in the GC [10]. This restricted expression of AID further supports the notion that eBL originates in the GC.

eBL is also closely associated with two infectious agents, *P. falciparum* malaria and Epstein-Barr virus (EBV) [1], [11], [12]. The distribution of the tumor in Africa closely matches that of hyper- and holoendemic malaria [12] while EBV was originally discovered in eBL tumor biopsies. Subsequently, we have learned a great deal about the molecular mechanism behind eBL pathogenesis and the transforming ability of EBV. EBV is a B lymphotropic herpes virus that can drive the activation and proliferation of newly infected B cells by expressing a series of latent proteins and noncoding RNAs that collectively are referred to as the growth transcription program[13], [14]. *In vivo,* however, EBV establishes a lifelong, quiescent, persistent infection in resting memory B cells [15], [16]. The virus makes the transition *in vivo* from a newly infected activated B cell blast to a resting memory B cell via passage through the GCs of the tonsillar lymphoid tissue [[17], [18]. In doing so it recapitulates the mechanism by which normal B cells become memory B cells (for a detailed description of the mechanism see [14]).

Normally deregulation of c-myc expression such as is found in eBL would lead to apoptotic death of the cell; however, evidence suggests that exposure to the EBV growth program prior to entry into the GC [14], [19] and viral genes expressed in the GC [20], [21] are sufficient to convey a level of resistance to this apoptosis. Thus, the cells in the GC most likely to tolerate the c-myc translocation are the ones already latently infected with EBV. This also places EBV at the site of eBL origin, the GC. Interestingly GC cells carrying EBV express only a limited subset of the latent proteins (default transcription program) and even these become silenced as the infected cells enter the memory compartment [16], [17], [22]. Here the virus only expresses small non-coding RNAs including ∼40 miRNAs. The exception is that they also express the viral DNA tethering protein EBNA1 when the cells occasionally divide as part of normal memory B cell homeostasis [23]. Viral gene expression in eBL resembles the infected dividing memory B cells, not the GC cell: i.e. viral gene expression is limited to the viral DNA tethering protein (EBNA1) and the non-coding RNAs. This led us to propose that eBL is a tumor of a GC cell that has left the lymph node to become a resting memory B cell but is unable to do so because it continues to proliferate, driven by the deregulated c-myc oncogene.

While understanding the function of c-myc and EBV in eBL has progressed, the role of *P. falciparum* malaria has remained poorly understood. *P. falciparum* malaria is immunosuppressive [24] and there is considerable evidence that this leads to much higher viral burdens of EBV [25], [26]. However it is well documented that increased EB viral loads associated with immunosuppression [27] predispose to EBV positive immunoblastic lymphoma not Burkitt's lymphoma [28]. We hypothesize that malaria plays multiple roles in eBL pathogenesis. First, we propose that malaria has the capacity to induce deregulated expression of AID thereby increasing the likelihood of the translocation event. Second, we suggest that the higher viral burdens lead to an increased frequency of newly infected B cell blasts in the tonsils. This results in more EBV infected B cells transiting the GC and consequently a higher frequency of cells in the GC able to tolerate a c-myc translocation. Taken together malaria infection would both increase the likelihood of c-myc translocations in the GC and the probability that it will occur in a cell that can tolerate it, namely an EBV infected cell. In this paper we have sought to test this hypothesis.

## Results

### Malaria parasites induce AID in B cells *in vitro*

**Figure 1:**
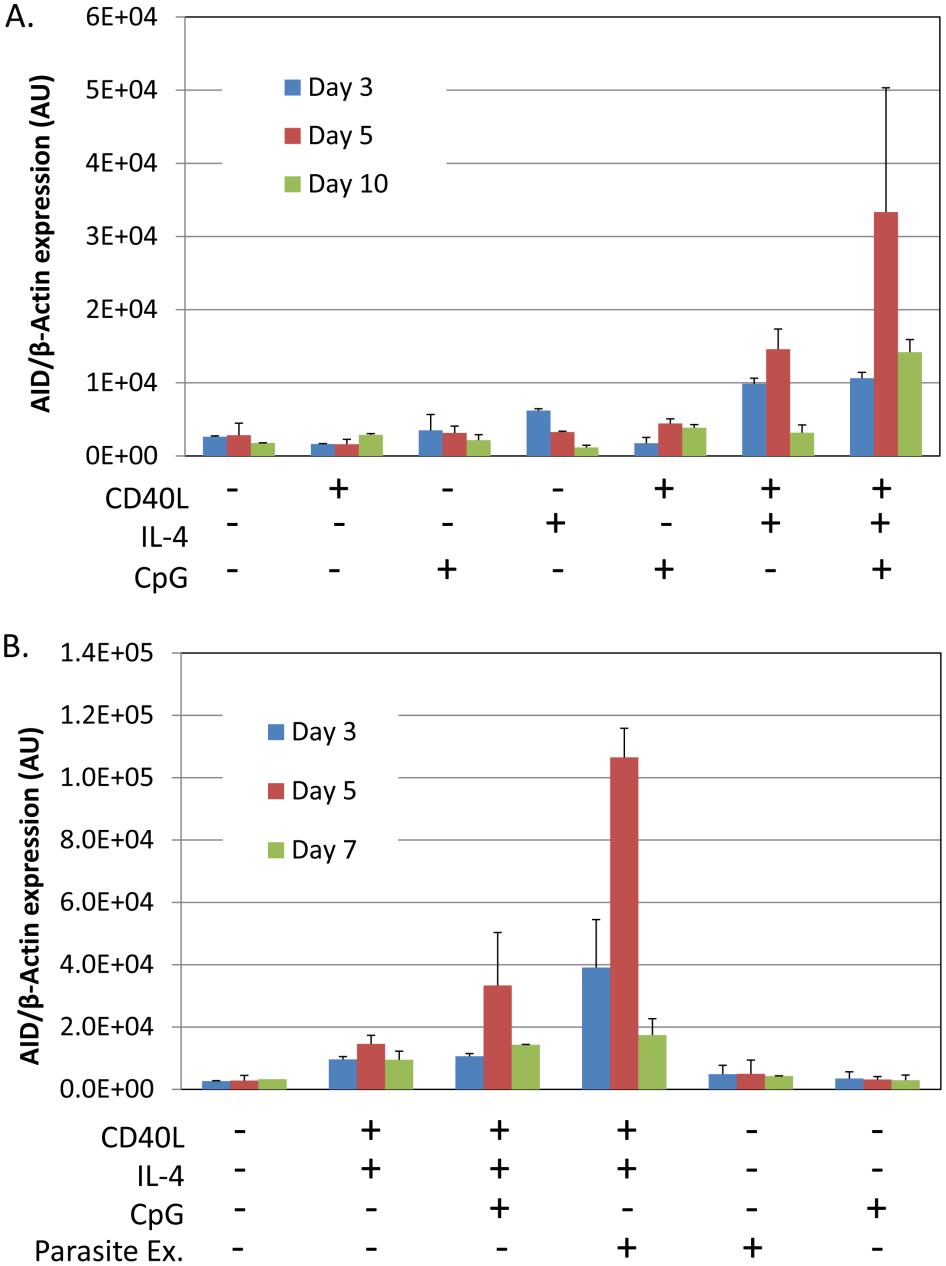
*P. falciparum* extracts stimulate expression of AID mRNA in normal tonsil B cells. Tonsils lymphocytes were isolated and incubated with the components indicated in the Figure for 3 (blue), 5 (red) or 7 (green) days. After the noted incubation times, B cells were isolated and analyzed for AID mRNA expression. A. Induction of AID mRNA requires a combination of signals from IL-4, CD40 ligand and CpG. B. *P. falciparum* extracts stimulate AID mRNA expression more effectively than CpG. AU – arbitrary units. The level of AID mRNA is expressed relative to that of β-actin.

To provide direct support for our hypothesis we have sought *in vitro* evidence that P. *falciparum* can stimulate AID expression. Tonsil B cells were incubated with malaria extract prepared by lysing red blood cells infected with *P. falciparum*. As controls we used the known Toll like receptor 9 (TLR9) agonist CpG and costimulation with IL-4 and CD40 ligand. Figure 1A shows a time course of AID mRNA induction with various combinations of stimulants. It is apparent that optimal induction requires a combination of T cell help (CD40 ligand and IL-4) and the TLR9 agonist CpG, with peak activation occurring after 5 days of culture. In our hands, CpG alone had minimal or no effect on AID induction although it was extremely potent in driving B cell proliferation (not shown). These results are consistent with previous reports [29]. Figure 1B shows the same experiment where extracts from lysed red blood cells infected with *P. falciparum* were also tested. Similarly to CpG, the parasite extract had minimal or no effect when added alone but showed strong stimulation of AID expression in combination with IL-4 and CD40 ligand. This suggests that the parasite would only stimulate AID expression when T cell help is available, i.e. in the GC. As with CpG activity peaked by day 5. At this time, the parasite extract was three times as effective as CpG in inducing AID mRNA. The parasite extract differed from CpG in that when added alone it was not able to stimulate B cell proliferation (not shown).

**Figure 2:**
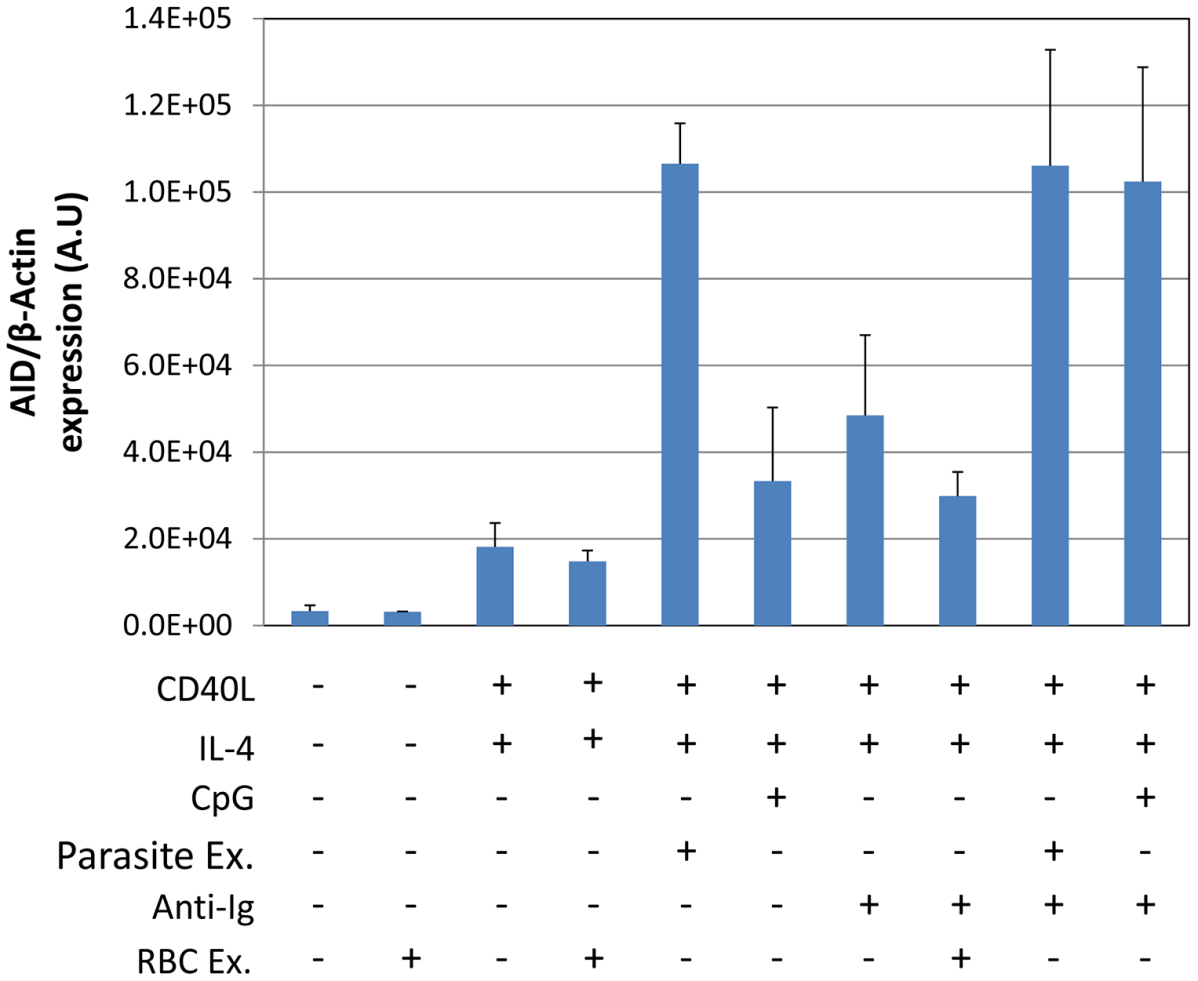
*P. falciparum* extracts stimulate AID expression at levels equivalent to CpG combined with surface Ig cross-linking. The assay was performed after incubation for 5 days as described in Figure 1.

Optimal stimulation of AID expression by CpG requires costimulation through the BCR. This is demonstrated in Figure 2, where inclusion of sIg cross-linking increased stimulation two and a half fold compared to CpG alone. Comparison to parasite extract in the same experiment demonstrated that sIg cross-linking plus CpG were about as effective as parasite extract alone and that sIg cross-linking did not significantly enhance the effect of parasite extract. Extracts from uninfected RBCs showed no activity either alone or in combination with other stimuli (Figure 2). We conclude that the parasite is able to generate a signal that is as effective as the TLR9 and BCR signals combined.

**Figure 3:**
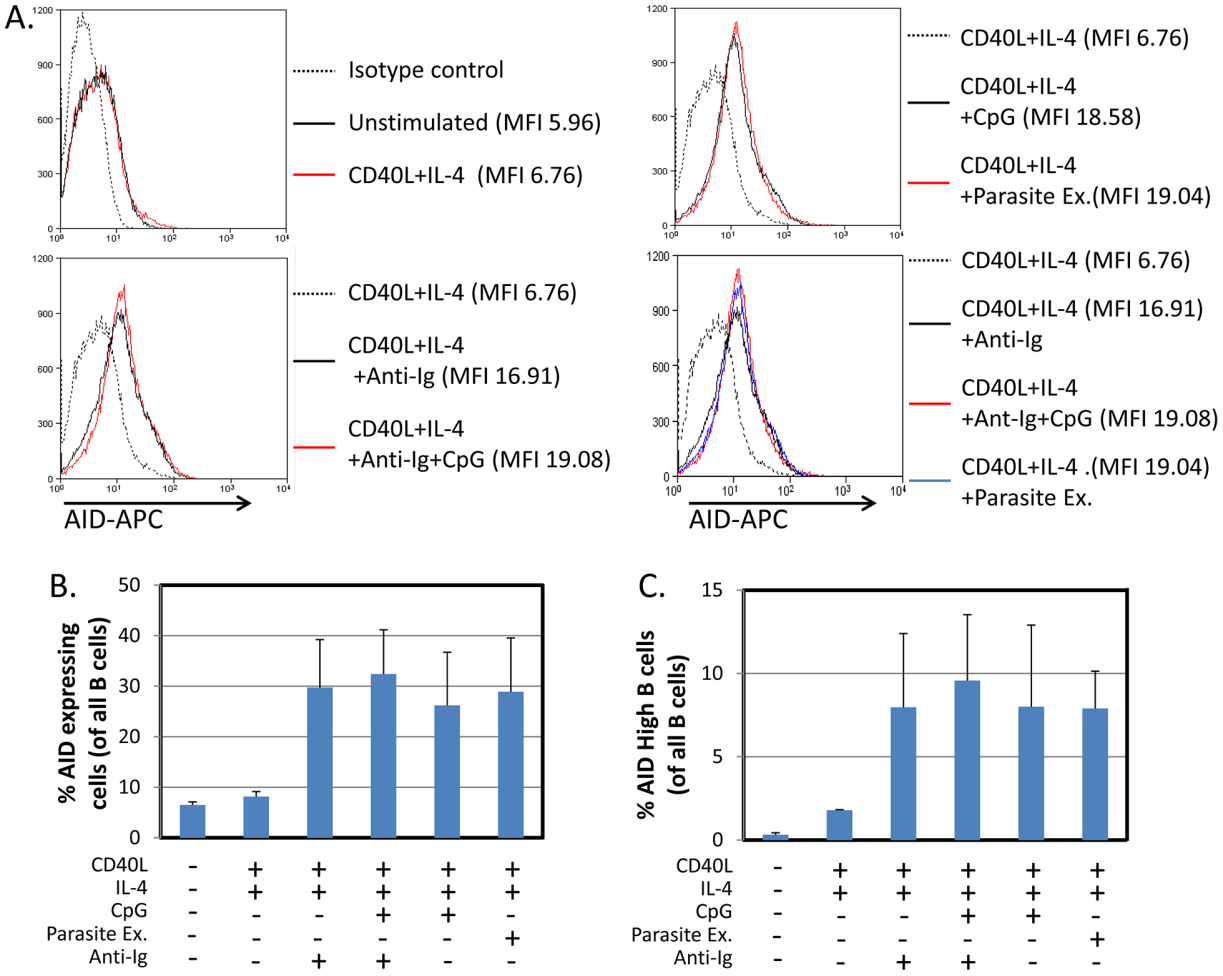
*P. falciparum* extracts stimulate expression of AID protein in normal tonsil B cells. The same protocol was followed as described in Figure 1, except the cells were analyzed by staining for AID protein expression and FACS analysis after a 5 day incubation period. - Flow cytometry histograms of cell populations demonstrate equivalent levels of AID expression in B cells activated by CpG or *P. falciparum* extracts. MFI  =  Mean fluorescence intensity. A. and C. Percentage of all B cells expressing AID (B) and percent of B cells expressing high levels of AID (C) suggest equal levels of AID expression in B cells activated by CpG or *P. falciparum* extract. We observed two overlapping populations of cells staining positive for AID. An arbitrary gate imposed on the brighter population defined high level expressers. The same gate was applied to all samples.

To confirm that the increase in AID mRNA stimulated by the parasite was reflected in increased AID protein expression, we repeated the stimulation experiments and examined the resulting cells for AID by flow cytometry. As may be seen in Figure 3A–C, parasite extract induced comparable levels of AID protein expression to that achieved with CpG when combined with sIg cross-linking. It is noteworthy that in some cases the relative level of protein expression measured by FACS did not match the levels seen for the mRNA (see for example Unstimulated versus CD40L +IL4). This likely reflects that the cells are newly stimulated in culture and there is a lag between the synthesis of AID mRNA and the production of the protein.

We conclude, therefore, that *P. falciparum* is a potent antigen independent stimulator of AID expression. When combined with T cell help signals it is at least as effective as the combination of CpG and BCR cross-linking.

### Hemozoin is an AID agonist

**Figure 4:**
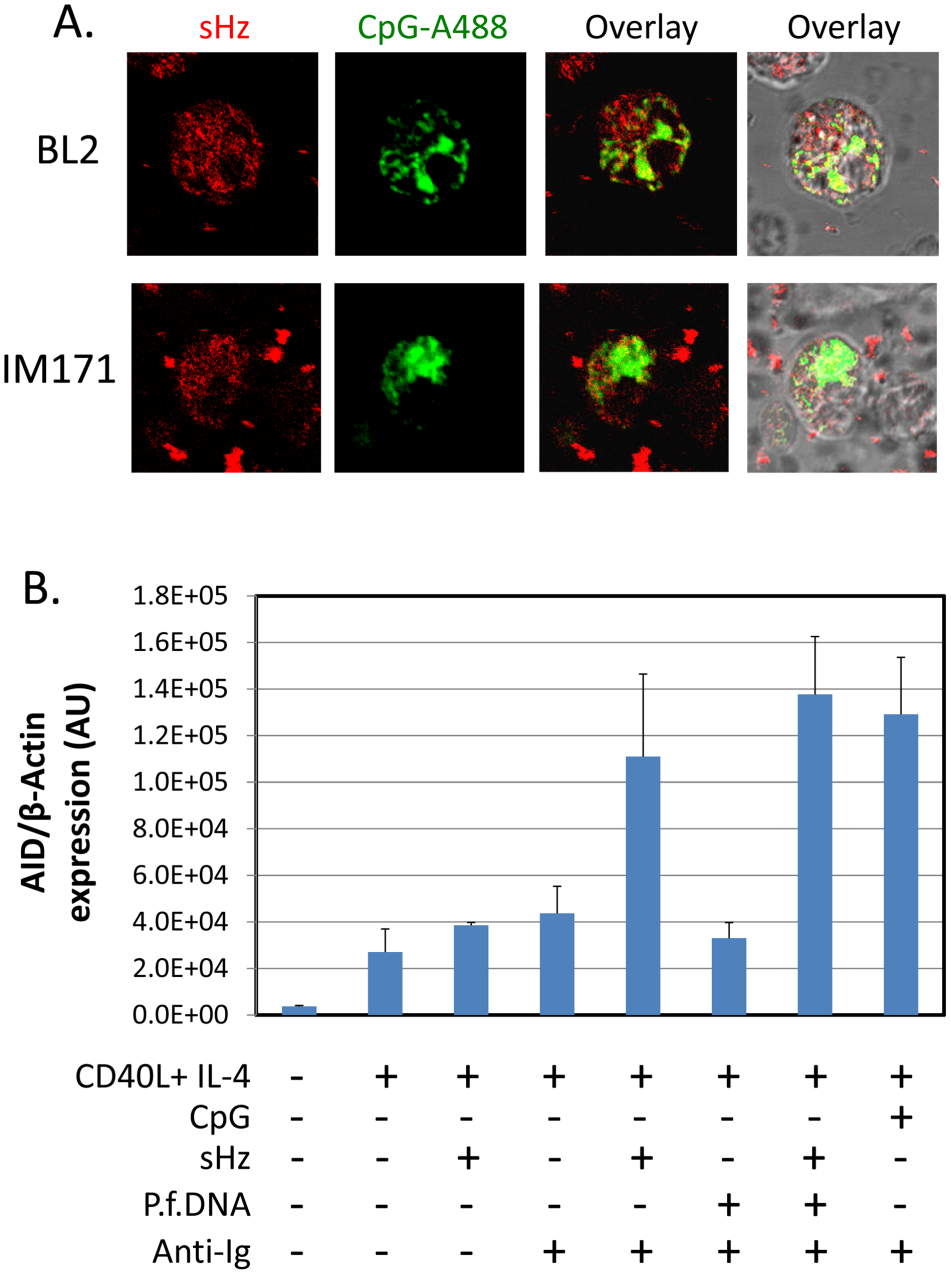
Hemozoin is taken up by B cells and activates AID expression. A. Hemozoin (sHz) (red) and CpG (green) were incubated with two different B cell lines. Note presence of red granules inside both cell types. BL2 is an EBV negative BL line and IM171 a spontaneous EBV positive lymphoblastoid line. B. Hemozoin stimulates expression of AID mRNA to an equivalent level to that obtained with surface Ig cross-linking and CpG. The stimulation was independent of hemozoin being complexed with parasite DNA. The same protocol was followed as described in Figure 1. The cells were analyzed after a 5 day incubation period.

It has been reported that the *P. falciparum* metabolic product of hemoglobin digestion, hemozoin [30], is a ligand for TLR9, but this has only been tested with dendritic cells [31], [32]. It has not been shown for B cells. To test if hemozoin could be taken up by B cells we have incubated hemozoin and CpG with two B cell lines, BL2 (EBV negative BL line) and IM171 (spontaneous EBV positive lymphoblastoid cell line). As may be seen in Figure 4A, both were readily taken up by the B cells (Hemozoin crystals were visualized using reflection microscopy and CpG is tagged with Alexa-488). To test if hemozoin could thereby act to stimulate AID expression we have compared the stimulatory activity of hemozoin to CpG and sIg. The result is shown in Figure 4B. Hemozoin alone had no effect, however in combination with sIg cross-linking it was two and a half fold more effective than sIg alone, comparable with CpG plus sIg. No appreciable increase was observed when parasite DNA was added (3 ug/ml) either alone or together with hemozoin. This suggests that the hemozoin preparation alone was sufficient for the activity.

These results demonstrate that hemozoin is one component of the *P. falciparum* extract that is capable of stimulating AID expression however there must be another component that is mimicked by sIg cross-linking to obtain the optimal stimulation obtained with whole parasite extracts.

### Higher levels of AID expression in GCs of malaria tonsils

**Figure 5:**
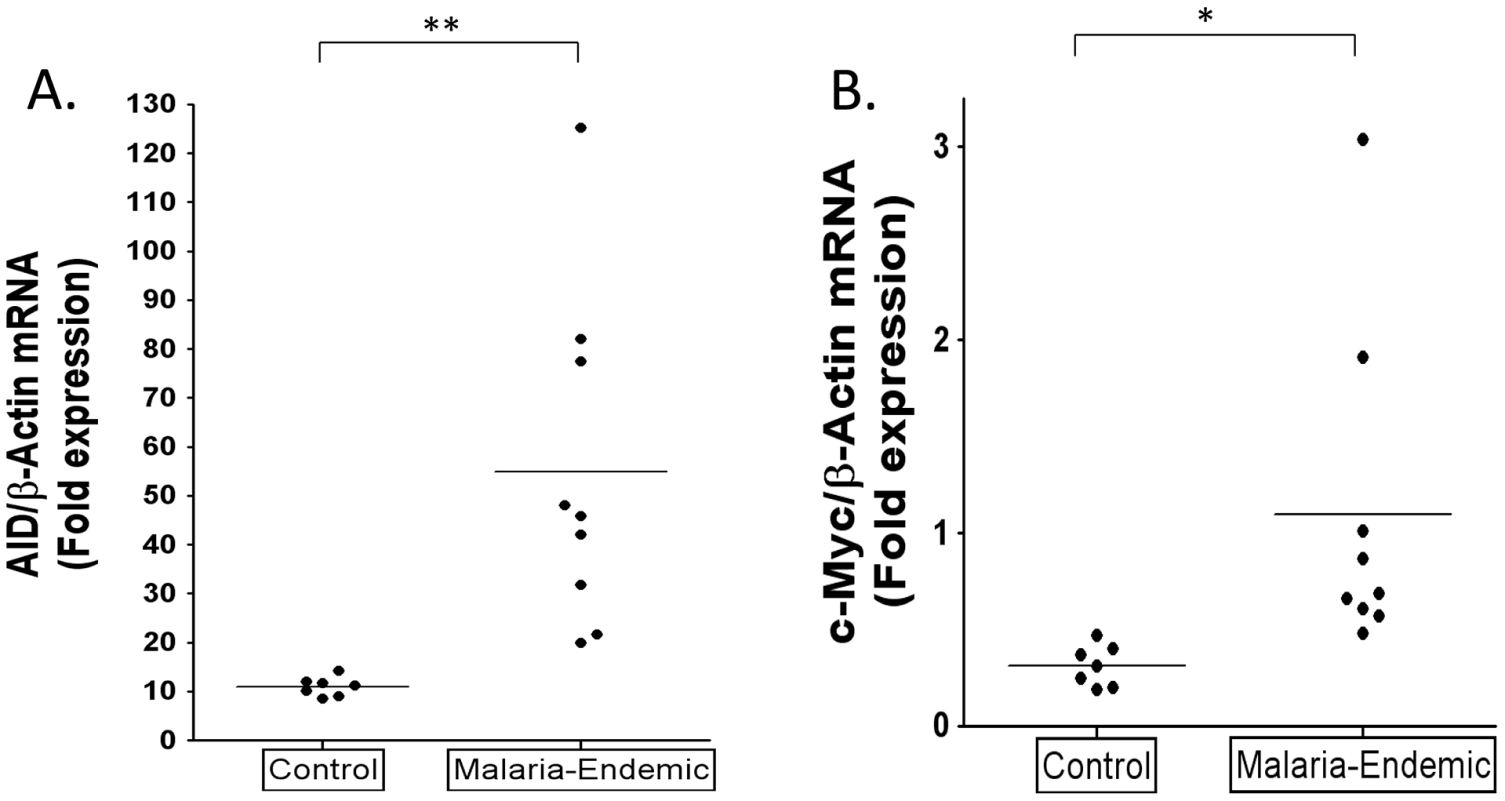
Higher levels of AID and c-myc mRNA in tonsil GC B cells from individuals infected with *P. falciparum* malaria compared to controls. A. AID mRNA expression is significantly increased in GC B cells from the malaria tonsils. (** p = 0.005). B. c-myc RNA is significantly elevated in GC B cells from some malaria tonsils. (* p = 0.031). AID and c-myc mRNA levels were normalized to β-actin and the level in the GC population is expressed relative to that in a standard naïve B cell population (calibrator) isolated from a single Boston tonsil.

To test the hypothesis that malaria is associated with higher levels of AID expression in vivo, we have examined and compared purified GC cells from tonsils obtained from malaria infected and uninfected patients who were matched for age, sex and socioeconomic class. The results are summarized in Figure 5A–B. mRNA was isolated from similar numbers of GC B cells. For all samples AID and c-myc mRNA levels were normalized to β-actin and the level in the GC population is expressed relative to that in a standard naïve B cell population (calibrator) isolated from a single Boston tonsil. The level of AID mRNA in the GC cells from the control population are comparable with what has been reported previously [33]. In comparison, the levels of AID mRNA in the malaria tonsils were significantly higher, about 5 fold on average. However, although all the values for the malaria tonsil were above the range of the controls, the spread was large such that some samples had levels 8–13 fold higher than controls. The results for c-myc transcripts were less striking. Most of the malaria samples showed little or no significant difference from controls with the exception that two of the nine samples tested clearly had a markedly elevated level of c-myc mRNA. The level of c-myc mRNA did not correlate with the levels of AID mRNA. These results confirm our prediction that the levels of AID expression are higher in the tonsil GC B cells of individuals infected with malaria.

### Higher levels of GC cells and EBV infected GC cells in malaria tonsils

**Figure 6:**
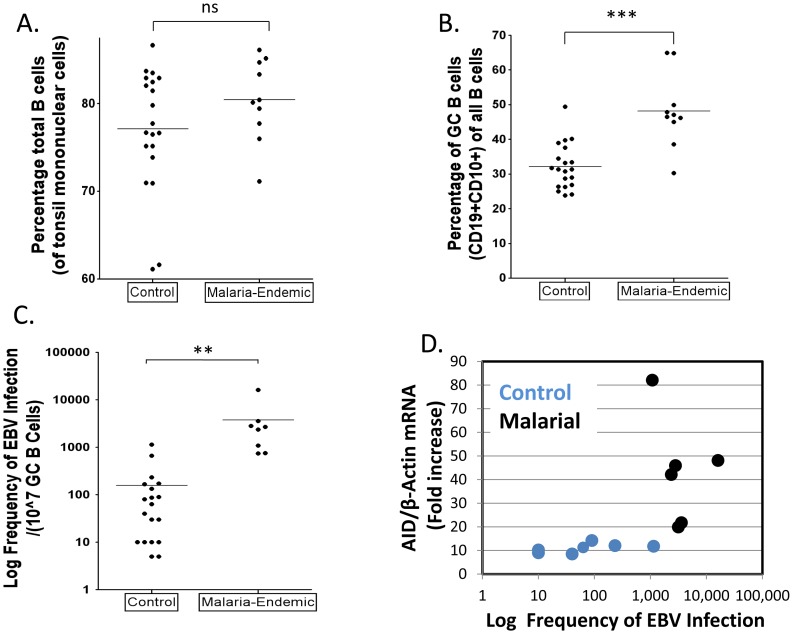
Higher levels of EBV infected cells in tonsil GCs from individuals infected with *P. falciparum* malaria compared to controls. A. The percentage of B cells (CD19+) is unchanged. (ns p = 0.18). B. The percentage of GC B cells (CD10+) is significantly elevated in the malaria tonsils (*** p<0.001). C. The frequency of GC B cells latently infected with EBV is dramatically increased in the malaria tonsils (*** p = 0.001). For details see Table 1. D. The level of EBV infected GC B cells and AID expression do not directly correlate.

To test the hypothesis that malaria is associated with higher levels of EBV infected cells in the GC, the GC cells from the same sets of tonsils described above were also analyzed for the presence of EBV. We recovered similar numbers of cells from both sets of tonsils and the fraction of B cells was also similar (Figure 6A). The fraction of GC cells in the control tonsils was ∼32% (Figure 6B), consistent with what we have seen in previous studies. However, the frequency in the malaria tonsils was unusually high ∼50%. When we analyzed the frequency of EBV infected cells in the two sets of tonsils, an even more dramatic difference was observed (Figure 6C and Table 1– note the log-scale in the Figure), with the level being ∼50 fold higher on average in the malaria tonsils compared to controls (log mean: 2,275/10^7^ versus 51/10^7^; median 2,580/10^7^ versus 52/10^7^). Taking the increased numbers of GC cells into account, this means that the malaria tonsils have approximately 70 fold more EBV infected cells in their GCs. These results confirm our prediction that the levels of EBV infected GC B cells should be higher in the tonsils of individuals infected with malaria.

**Table 1:** The frequency of GC B cells latently infected with EBV in tonsils from an area of holoendemic malaria and uninfected controls.

Malaria	Frequency of EBV Infected Tonsil GC Cells/10^7^ *								Mean
1	3,420	5,030	2,820	2,210	4,025	3,220	2,210		3,152
2	3,200	2,240	2,020	5,030	4,025	5,030	4,030	3,020	3,574
3	805	2,010	500	1,005					1,080
4	705	905	600						737
5	605	905							755
6	16,090	18,105	15,090	15,090					16,094
7	2,020	2,700							2,360
8	2,800								2,800
								log Mean	2,276
								Median	2,580
Control									
1	100	90	85	85					90
2	200	160	170	155					171
3	40								40
4	80	100	80						87
5	0	0	0						0
6	1,005	1,410	1,005						1,140
7	30								30
8	30								30
9	5								5
10	805	500	700						668
11	200	300	200						233
12	10								10
13	80								80
14	100	200	200						167
15	80	60	50						63
16	100	200	100						133
17	5								5
18	10								10
19	10								10
20	10								10
21	0	0							0
								log Mean	51†
								Median	52

* - values represent separate estimates made on the same tonsil at different times.

† - NB. This is an over estimate since it does not include samples 5 and 21 where no EBV infected cells were detected.

As may be seen in Figure 6D, although both EBV infection and AID expression were elevated in all of the malaria tonsils there was no correlation between the levels of EBV infection and the level of AID in either malaria or control samples. We conclude that individuals infected with malaria have an increased level of EBV infected cells and AID mRNA expression in their tonsil GC cells, but these levels are not correlated.

### c-myc expression in the GC

**Figure 7:**
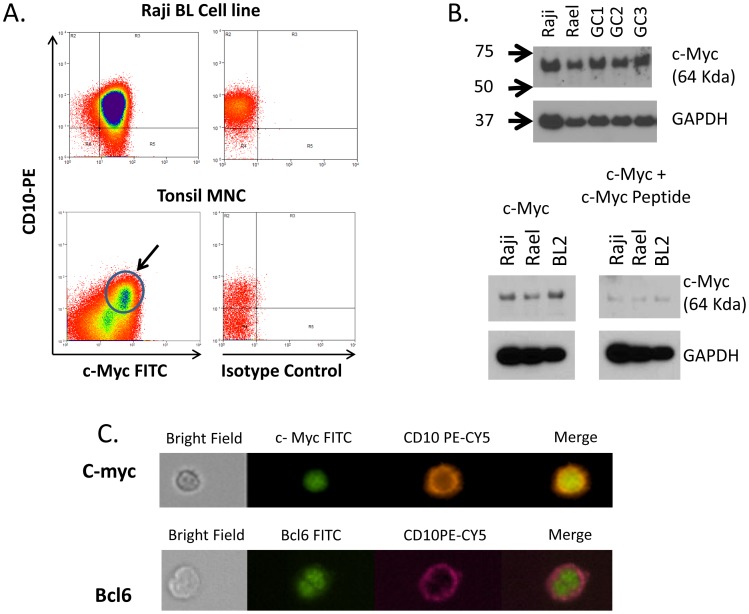
c-myc is expressed in GC B cells. A. Flow cytometric analysis demonstrating c-myc expression in tonsil GC cells (CD10+). The arrow indicate the CD10+, c-myc positive GC population. B. Western blot analysis confirming c-myc expression in 3 independent tonsil GC B cell preparation (GC1-3). Raji and Rael are EBV positive BL cell lines. The molecular weight in KD is shown to the left. C. ImageStream analysis of c-myc positive tonsil GC B cells. Staining for a known nuclear protein bcl-6 is shown for comparison. N.B. For this study only Boston control tonsils were used.

We have shown above that the level of c-myc transcripts is significantly elevated in GC cells from the tonsils of a subset of individuals with malaria. However, there has been controversy as to whether c-myc is actually expressed in the GC [34], [35], [36], [37]. Therefore, to confirm that we were detecting c-myc expression in GC cells we performed several experiments as shown in Figure 7. GC cells (CD10+ tonsil B cells) were positive for c-myc expression when stained with a c-myc specific antibody and analyzed by flow cytometry (Figure 7A). The presence of c-myc protein in our GC cell preparations was confirmed by Western blot (Figure 7B), where the signal was specifically blocked by the myc specific peptide used to raise the antibody. The specificity and correct location of the protein in the nucleus was confirmed by analysis with the ImageStream (Figure 7C). We conclude, therefore, that our studies support the current opinion that c-myc is expressed in GC cells.

## Discussion

It is now more than 50 years since the association between *P. falciparum* malaria and eBL was first proposed [11], [12]. Since that time, confirmation of a direct link and a mechanism to explain it has been lacking. Here we have presented studies on the effect of malaria in the context of the GC and provided evidence for a multifactorial effect of malaria that can account for the increase risk of eBL. These include the activation of AID expression, possible heightened c-myc transcription, increasing the numbers of B cells transiting the GC and increasing the fraction of these cells that are EBV infected. The common component linking these effects is the GC. The GC is the structure where immunoglobulin genes undergo somatic hypermutation and class switch recombination [38], [39], mediated by AID [10], as the cells undergo affinity maturation. It is this enzyme that is responsible for causing the c-myc translocation characteristic of eBL [7], [8], [9]. However, the GC is also the site where newly infected EBV blasts undergo the transition to become resting latently infected memory B cells [14], [16], [22]. Therefore, in the presence of malaria the number of cells able to tolerate a c-myc translocation (EBV infected) in the GC are increased and the probability of a c-myc translocation is also increased (AID activation). Thus, it is the increased probability of a fortuitous collision of AID and EBV in GC cells, both exacerbated by malaria that leads to eBL. A further observation confirming that these events occur within the GC was the finding that the *P. falciparum* extract alone had little or no ability to induce AID. Maximal induction required co-stimulation with CD40 ligand and IL-4. This means that the malarial parasite can only work to induce AID expression if T cell help is also provided. Since T cell help is specifically provided in the GC [40], [41], this firmly places the role of *P. falciparum* in the induction of AID in the GC and would seem to rule out fortuitous activation elsewhere. This would explain why eBL is uniquely a tumor of GC cells.

We observed no difference in the percentage of total B cells per tonsil between the malaria-endemic and the non-malaria regions, however, GC cells are ∼2 fold higher in the malaria background. This suggests that malaria (or malaria background) does not disrupt the size of the B cell pool but increases the likelihood that more B cells, and therefore EBV-infected cells, either enter the GC or that the cells stay longer in the GC. Either way, this increases the chances that some B cells, including EBV infected cells, will develop deleterious mutations and translocations. In individuals with malaria we detected a wide range in the number of GC cells containing EBV and in the degree of AID expression. It is tempting to speculate that those with the rare fortuitous coincidence of extremely high levels for both may be the likely candidates for tumor development.

We have provided compelling *in vitro* evidence to support the claim that *P. falciparum* induces AID expression, the first such evidence. Importantly, the parasite induces AID in an antigen independent, i.e. deregulated fashion. Such expression is known to be a predisposing factor for the c-myc translocation. EBV infection of B cells *in vitro,* as well as EBV associated proteins, have also been shown to induce AID expression [42], [43], [44]. It is conceivable therefore that EBV and malaria could even cooperate in the induction of AID.

We have shown that *P. falciparum* is capable of eliciting AID expression at least as effectively as the combination of CpG and BCR cross-linking, suggesting that the parasite can provide both signals. We have identified hemozoin, the crystalline by product of *P. falciparum* digestion of hemoglobin [30] and a reported TLR9 ligand [31], [32], as one of the parasite components responsible. Consistent with its signaling through TLR9, hemozoin is only effective at inducing AID in the presence of BCR cross-linking. Thus, it is likely that there is a second, as yet unidentified, parasite derived ligand that provides the surrogate BCR signal. A likely candidate for this is the *P. falciparum* specific protein PfEMP1 encoded by the *var* gene family [45], which has been shown previously to bind to and activate B cells through the BCR [46]. This would also explain the specific link of *P. falciparum* with eBL since only this species of malaria expresses PfEMP1. The prediction is therefore, that a combination of hemozoin and PfEMP1 are providing the requisite TLR9 and sIg signals. Since TLR9 is a receptor for polynucleotides it was somewhat surprising that hemozoin was able to stimulate AID expression in the absence of parasite DNA. It is controversial as to whether hemozoin does [32] or does not [31] need or be associated with DNA in order to signal through TLR9. It is possible that hemozoin may be signaling via a TLR9 independent mechanism in our system since it has been reported that it can signal through other pathways including the inflammasome [47], [48]. However, there was a high death rate in our in vitro assays therefore it is possible that endogenous unmethylated DNA from the dead cells was being trafficked into the endosomal compartment by hemozoin to stimulate TLR9 and consequently lead to AID induction.

Demonstration of an increased viral burden of EBV in individuals with *P. falciparum* malaria is not a new observation [25], [26], however in this paper we have shown a direct mechanistic consequence of this elevation that provides a risk factor for eBL namely an increased number of latently infected cells in the GC. This effect is not modest, with the mean frequency of EBV infected GC B cells from the malaria tonsils being about 50 fold more than the frequency of EBV infected GC B cells from the controls. Combined with the increase in the number of total GC cells this results on average in there being 70 times more EBV infected cells in the GCs of individuals with malaria compared to controls. We have shown previously that healthy individuals have at any time on average approximately 3 EBV infected cells per GC [49]. Our results here indicate that this would increase to around 150–200 per GC for an individual with malaria. This is a significant increase in risk since the consequence is an extremely high number of (EBV infected) cells each of which are primed to tolerate the c-myc translocation that emanates from overexpressed AID.

The increased frequency of latently infected cells seen in the malaria samples (∼50 fold) is very similar to what we have reported previously for immunosuppressed patients and patients with SLE [27], [50]. Indeed, it has been reported previously that malaria is immunosuppressive for T cell responses [51], including those directed against EBV [24]. It is tempting therefore to speculate that children with malaria are immunosuppressed and that this explains the risk for eBL. However, we have pointed out previously that immunosuppression is a risk factor for post-transplant lymphoproliferative disorder (PTLD)-like disease, i.e. immunoblastic lymphoma not eBL [14], [22]. Thus, immunosuppression alone is not sufficient to explain eBL.

The results presented here also provide further support for the GC model of EBV persistence. This model, which is now generally accepted, holds that EBV establishes a persistent infection by driving newly infected blasts through the GC to become latently infected resting memory B cells [14], [16], [22]. A direct prediction of this model is that a higher viral burden should produce a higher number of EBV infected cells transiting the GC and that prediction has been fulfilled in this study. Furthermore, the studies presented here provide a powerful functional confirmation of the model in that they provide an explanation for the link between malaria and eBL. Thus, the model predicts that it is the elevated rate of passage of virus infected cells through the GC, together with deregulated AID expression, that explains the origin of eBL.

Our studies suggest that malaria may also induce heightened expression of c-myc in the GC, at least in some individuals. c-myc is a non-traditional transcription factor with a very complex regulation. High c-myc expression in the GC could account for the observed high rate of proliferation, as well as the high apoptotic tendency of GC B cells [34], [37]. However, the question of whether c-myc is even expressed in the GC has been controversial in the past. Martinez-Valdez et al. [37] and Cutrona et al. [34] report that c-myc is highly expressed in GC B-cells, whereas Klein et al. [36] were unable to confirm these findings. Recently, Dominguez-Sola et al. [35] have presented compelling evidence that c-myc is expressed in GC cells, specifically by B cells selected for reentry into the dark zone, a conclusion supported by our work. Furthermore, for AID to target c-myc in GC B cells, c-myc must be expressed since AID deaminates transcribed substrates and acts on selected highly transcribed genes when they are over-expressed. Thus, higher than normal levels of c-myc transcription driven by malaria could further increase the risk that AID would target the c-myc gene for a translocation event.

In conclusion, we have presented the first direct evidence for a mechanism to explain the link between eBL and holoendemic malaria. If correct, these observations imply that reducing the exposure to *P. falciparum* malaria or the development of drugs to block the ability of malaria to induce AID should dramatically reduce the incidence of eBL in young children in tropical Africa. Specifically this should act as a spur to agencies interested in reducing the incidence of exposure to *P. falciparum* in young children.

## Methods

### Ethical statement

This study was approved by the Institutional Review Board of the Tufts Medical Center, Boston, USA and the Committee on Human Research and Ethical Publications of the School of Medical Sciences, Kwame Nkrumah University of Science and Technology (KNUST), and Komfo Anokye Teaching Hospital (KATH), Kumasi Ghana. The material used was deidentified, discarded tonsil tissue and was deemed exempt from informed consent by the IRB. The tonsil material was obtained indirectly either through the Pathology Department at Tufts Medical Center or the EENT Clinic at Komfo Anokye Teaching Hospital.

### Cells, cell lines and tissues

The EBV-positive lymphoblastoid cell line IB4 (gift of Elliott Kieff) and Namalwa Burkitt's lymphoma cell line were used as positive controls for DNA PCR of the W-repeat region of the EBV genomes. The EBV-negative cell line CB60, a mouse T-cell hybridoma cell line (gift of Miguel Stadecker) was used as a negative control in all W-PCR experiments. The Burkitt's lymphoma cell lines Raji and Rael were used as positive controls for c-myc western blot. The EBV negative BL2 Burkitt's lymphoma cell line and SP-IM 171 spontaneous EBV lymphoblastic cell line were used in hemozoin-DNA complex internalization assays. All cell lines were cultured at 37°C with 5% CO_2_ in RPMI 1640 supplemented with 10% fetal bovine serum, 2 mM glutamine, 2 mM sodium pyruvate, 100 IU of penicillin-streptomycin, and 10 µg/ml ciprofloxacin hydrochloride (RPMI-complete). Palatine tonsils were obtained from patients 14 years or younger undergoing routine tonsillectomy. Twelve tonsil samples were obtained from patients at the EENT Clinic Komfo Anokye Teaching Hospital, Kumasi, Ghana. These were processed at the Kumasi Center for Collaborative Research in Tropical Medicine (KCCR), Kumasi, Ghana, stored in liquid nitrogen and shipped to Tufts University School of Medicine, Boston, USA on dry ice (under the supervision of Prof. Karen Duca, KNUST, Kumasi, Ghana). The presence of the parasite was confirmed based on detection of *P. falciparum* DNA and/or antigens (see below). Kumasi is an area of holoendemic malaria therefore it was not surprising/unexpected that all patient samples received tested positive for the parasite. To obtain parasite free control samples we therefore also collected twenty one tonsils from a malaria negative region (Boston MA) through the Pathology Department at Tufts Medical Center, Boston MA, USA. The identical procedure and reagents were used for harvesting tonsils in Boston and Kumasi. The malaria infected individuals who provided the tonsils are genetically distinct from the controls and could be subject to a wider range of infection and lower level of general hygiene. To minimize this possible source of variation we collected the malaria tonsils from age and sex matched donors in an area of high socioeconomic status in Kumasi where economic and medical standards were comparable to those in Boston.

### Isolation of tonsil mononuclear cells

Tonsil tissue was cut into very small pieces in ice-cold PBSA (1x PBS +0.5% BSA) and then minced. Supernatants were pipetted through a cell strainer into 50 ml conical tubes to remove debris. Supernatants were centrifuged at 1,600 rpm at room temperature for 10 minutes and then aspirated. Pellets were re-suspended and brought to 50 ml with PBSA. About 25 ml of cells was carefully layered onto 20 mls of Ficoll-paque plus (GE Healthcare Biosciences, Philadelphia, USA) and then spun at 2,000 rpm for 30 minutes at room temperature (with no brake). Mononuclear cells were collected from the interface (buffy coat) and cell pellet discarded. The volume of mononuclear cells was adjusted to 50 ml with PBSA (and an aliquot taken for counting); cells were then washed once by spinning at 1,500 rpm for 10 minutes. After counting, cells were frozen in fetal bovine serum (FBS) (Sigma, St. Louis, USA) plus 10% DMSO (dimethyl sulfoxide) at 1×10^8^ cells/ml. Cells were aliquoted in cryotubes and kept on ice for about 5 minutes, stored at −80°C overnight and then transferred to liquid nitrogen for long term storage. The identical procedure and reagents were used for preparing tonsil cell suspensions in Boston and Kumasi.

### Extracellular and intracellular staining

Tonsil mononuclear cells were thawed in medium (RPMI/FBS), and spun down at 1,500 rpm for 5 minutes. B cells were either first purified using StemSep (StemCell technologies, Vancouver, Canada) according to manufacturer's instruction, or cells were re-suspended in 0.5% BSA in 1x PBS (PBSA) i.e. staining buffer. Cells were resuspended at 5×10^6^ cells/100 µl PBSA either directly into FACs tubes or 15 ml tubes for staining. For extracellular staining the appropriate concentration of fluorochrome conjugated antibody was added to cells in appropriate tubes and incubated for 15 minutes at room temperature in the dark, after thorough mixing. Cells were washed once with PBSA, vortexed, and spun down at 1,500 rpm for 5 minutes. Finally cells were re-suspended in 300 µl PBSA and stored at 4°C until analyzed.

For intracellular staining tonsil cells were pelleted, washed in Dulbecco's PBS^−^ (without calcium or magnesium), and fixed either with 4% formaldehyde or with BD Cytofix/Cytoperm fixation and permeabilization solution (BD Biosciences, San Jose, USA) and then incubated for 20 minutes at room temperature. Cells were then washed twice either with 1x BD wash/perm buffer (BD Biosciences, San Jose, USA) or with 0.04% saponin based wash buffer and spun down at 1,500 rpm for 5 minutes. Permeabilization buffer (0.5% saponin based buffer) was used to re-suspend cells at 5×10^6^ cells/100 µl. Two microliters (2 µl) of normal human serum (60 mg/ml, Thermo Fisher Scientific Rockford, USA) was added in order to block against non-specific antibody binding, and cells were incubated for 30 minutes at room temperature. Primary antibodies for intracellular antigens were added and incubated at room temperature for 30 minutes. Cells were then washed once and re-suspended in 0.5% saponin based buffer, 2 µl of normal human serum was again added and incubated for 30 minutes at room temperature. Fluorochrome conjugated secondary antibodies were then added at appropriate dilutions and incubated for 15 minutes in the dark. Cells were washed once with wash buffer, spun down at 1,500 rpm for 5 minutes and stored in 300 µl PBSA at 4°C until analysis. Cell sorting was performed on a MoFLo or Influx cell sorter and analysis on a FACSCalibur or Image Stream at Tufts University laser cytometry core. Sorted populations were >90% pure.

A list of the antibodies and fluors used in this study is given in Table S1.

### Detection of *P. falciparum* in tissues

The presence of the parasite was confirmed based on detection of *P. falciparum* DNA and/or antigens. Nested, parasite specific DNA PCR was performed for a sequence in the 2^nd^ exon of the Chloroquine transporter (*Pfcrt*) gene as follows: First round (94°C, 3 min; 94°C, 30 sec; 56°C, 30 sec; 60°C, 1 min 30 cycles, 72°C, 3 min) forward primer CCGTTAATAATAAATACACGCAG, reverse CGGATGTTACAAAACTATAGTTACC (95°C, 5 min; 92°C, 30 sec; 48°C, 30 sec; 65°C, 30 sec (25 cycles); 72°C, 3 min) forward primer TGTGCTCATGTGTTTAAACTT reverse ACAAAATTGGTAACTATAGTTTTG. PCR product sizes were verified on 2% agarose gels. 1^st^ round amplicon 527 bp, 2^nd^ round 145 bp.

*P. falciparum* antigens were detected employing a rapid diagnostic test (RDT) cassette (ACON Laboratories, Inc., San Diego, USA) as directed by the manufacturer.

### Limiting dilution analysis to measure the frequency of EBV infected cells

For limiting dilution analysis, GC B cell (CD19+CD10+) populations were isolated by flow cytometry and usually 10 replicates each of 2×10^5^, 1×10^5^, 5×10^4^, 2.5×10^4^, 1.25×10^4^, 0.625×10^4^, 0.3125×10^4^ (higher or lower dilutions were added as needed) were placed in a 96 V bottom plate for subsequent EBV W-repeat DNA PCR. The plate was spun at 1,200 rpm for 10 minutes at 4°C and the supernatant aspirated. To each well was then added 20 µl of digestion mix (10X PCR buffer, 100 µl; Igepal (NP-40) 100 µl; Tween-20 100 µl; Proteinase K 50 µl of 20 mg/ml stock and 650 µl of water). The plate was sealed air-tight and incubated at 55°C overnight. This was followed by proteinase K deactivation at 95°C for 10 minutes. Ten microliters (10 µl) of water was then added to all the sample wells. The fraction of EBV positive wells was then assessed by W-repeat EBV DNA PCR for each replicate and the frequency of EBV infected cells in the starting population calculated using Poison statistics. The EBV-positive cell lines IB4 and Namalwa were used as positive controls and the EBV-negative cell line CB60 was used as a negative control.

### W-repeat EBV DNA PCR

DNA real-time PCR specific for the W-repeat sequence of the EBV genome was performed as described [52]. For each reaction, a master mix was prepared, containing 12.5 µl IQ Supermix (Biorad cat 170-8862), 2.5 µl of 900 nM primers and 2.5 µl of 250 nM fluorogenic probe. Five microliters (5 µl) of DNA was added to 20 µl of master mix with a final reaction volume of 25 µl. (See Table S2 for primer and probe sequences). The PCR reactions were performed on a Biorad iCycler. The protocol was as follows: Step 1 (1 cycle): 3′ at 95°C; Step 2 (50 cycles): 15″ at 95°C, 1′ at 60°C.

### Purification of RNA and RT-PCR

RNA was purified by TRIzol extraction (Invitrogen, Life Technologies, Grand Island, USA) and then treated with TURBO DNase (Ambion, Life Technologies, Grand Island, USA) to eliminate DNA prior to RNA amplification (where necessary). cDNA was made from the RNA using a cDNA synthesis kit (Invitrogen iScript cDNA synthesis kit). For the cDNA synthesis reaction, a master mix was prepared which included 4 µl of 5X iScript reaction mix, 1 µl of iScript reverse transcriptase, and 8 µl of nuclease-free water. Seven microliters (7 µl) of purified RNA was added to 13 µl of master mix. All reactions were performed on an Applied Biosystems PCR machine (Thermal cycler). The protocol was as follows: one cycle that included 5 minutes at 25°C, 30 minutes at 42°C, and 5 minutes at 85°C. For real time PCR a master mix was prepared, containing 12.5 µl of IQ Supermix (Bio-Rad), 2.5 µl of 900 nM primers, and 2.5 µl of 250 nM fluorogenic probe, except when Taqman pre-developed assays were being used in which case, 12.5 µl Supermix, 1.25 µl 20x primer-probe mix and 6.25 µl water. Five microliters (5 µl) of cDNA was added to 20 µl of master mix with a final reaction volume of 25 µl. All real time PCRs were performed on a Bio-Rad iCycler. The protocol was as follows: step 1, one cycle of 3 minutes at 95°C; step 2, 55 cycles of 15 seconds at 95°C and 1 minute at 60°C. All our real time PCR assays were optimized to detect down to the single cell level (See Supplemental Information for a list of primers and probes).

### Preparation of *Plasmodium falciparum* extract

*P. falciparum* parasites (3D7 line) were cultured using standard procedures as described [53]. Parasites were grown at 5% hematocrit in RPMI 1640 medium, 0.5% AlbuMAX II (Invitrogen), 0.25% sodium bicarbonate, and 0.1 mg/ml gentamicin. Cultures were incubated at 37°C in an atmosphere of 5% oxygen, 5% carbon dioxide, and 90% nitrogen. Parasite extracts were prepared by selective lysis of the host RBC membranes through the addition of saponin. Infected RBCs were suspended in 0.01% saponin in PBS and incubated at room temperature for 5 min. Host cell free parasites were pelleted by centrifugation, washed twice with PBS and stored frozen at −80 degrees C. Parasite extracts were prepared by 3 freeze-thaw cycles of frozen parasites, sonicated and stored at −80 degrees Celsius until needed. The protein concentration was determined by the Bicinchoninic assay (BCA assay – see below).

### Stimulation assay

Fresh tonsil mononuclear cells (MNC) were isolated as described above and re-suspended at ∼1×10^6^ cells/ml in pre-warmed PBSA. Two microliters of 5 mM CFSE (carboxyfluorescien diacetate, succinimidyl ester) in DMSO (Invitrogen, Life Technologies, Grand Island, USA) was added per ml of cells (final concentration 10 µM CFSE). The cells were then incubated for 10 minutes at 37°C. Cells were spun down and re-suspended in 2 volumes of ice-cold culture medium and incubated on ice for 5 minutes. Cells were re-pelleted and re-suspended in 2 volumes ice cold culture medium (2 times). Washing was done one more time in 2 volumes of pre-warmed medium and cells were finally re-suspended in fresh pre-warmed cell culture medium (described above). A final concentration of 3 µM of CpG-2006 (Hycult Biotech, Plymouth Meeting, USA), 0.25 µg/ml CD40 ligand (eBioscience, San Diego, USA), 5 ng/ml IL-4 (eBioscience, San Diego, USA), 2.5 or 5 µg/ml Anti-human IgG+IgM, (Jackson ImmunoResearch, West Grove, USA) 10 µg/ml of crude parasite extract, and hemozoin (InvivoGen, San Diego, USA) 50 µg/ml were used in the tonsil mononuclear cell stimulation. Cells were harvested on days 3, 5, 7 and 10, stained with CD19 and then B cells were sorted with the MoFlo.

### Hemozoin internalization assay

Five microgram of human CpG-2006 DNA with a phosphorothionate (PTO) backbone bound to Alexa-488 (Integrated DNA Technologies, Coralville, USA) were mixed with 100 µg/ml of sonicated synthetic hemozoin [32]and co-incubated with rocking for 2 h followed by washing of the complex three times with PBS. Bound and unbound DNA were determined by measuring the DNA concentration in the collected supernatant, using the Nanodrop. BL2 and IM 171 cells were each plated at 1 ml (with approximately 500,000 cells per confocal plate). The CpG-Alexa-488-hemozoin complex was added to the cell lines BL2 and SP-IM 171, and incubated for 2 hours after which confocal microscopy was carried out. Confocal reflection microscopy for detection of hemozoin was combined with fluorescence microscopy to detect the Alexa-488 tagged CpG on a Leica SP2 AOBS confocal laser-scanning microscope as described in detail in [54].

### Western blot

Cells were spun at 1,500 rpm for 5 minutes and the supernatant was aspirated. 5×10^6^ cells were re-suspended in 100 µl of RIPA buffer (Sigma, St. Louis, USA) with freshly added protease inhibitors (Thermo Scientific, Rochford, USA). The sample was pipetted up and down to dislodge cell clumps, and then vortexed vigorously for 15 seconds then incubated on ice for ∼7 minutes, vortexed again for 15 seconds, spun at 13,200 rpm for 16 minutes at 4°C and the supernatant transferred to a new eppendorf. Protein concentrations were determined with either the Bio-Rad protein assay reagent (Bio-Rad, Hercules, USA) or the bicinchoninic acid (BCA) protein assay reagent mix (Thermo Scientific, Rockford, USA), according to manufacturer's instruction. To each protein sample was added 12.5% beta mercaptoethanol and 1× SDS sample buffer (Boston Bioproducts, Ashland, USA). Five microliters of protein standard (Biorad, Hercules, USA) was added to designated well(s). Samples were heated at 95°C for 5 minutes and resolved on 4–20% Tris-Glycine gels (Invitrogen, Life Technologies, Grand Island, USA). Immobilon-P polyvinylidene fluoride (PVDF) transfer membrane (Millipore, Billerica, USA) was used in a semi-dry electrophoretic transfer. The membrane was blocked with either membrane blocking solution (Invitrogen, Life Technologies, Grand Island, USA) or 5% milk in 1x Tris buffered saline with Tween 20 (TBST) (Cell signaling, Danvers, USA) at room temperature for 1 hr. The PVDF membrane was then incubated with primary antibody in appropriate blocking buffer at 4°C overnight. The membrane was washed in TBST or Invitrogen wash solution three times, 10 minutes each and incubated with secondary antibody in 5% milk in TBST for 45 minutes at room temperature. The membrane was then washed with Invitrogen wash solution 4 times, 10 minutes each at room temperature and then enhanced chemiluminescence (ECL) reagent SuperSignal west femto maximum sensitivity substrate (Thermo Fisher scientific, Rockford, USA) was applied to the membrane. Hyblot CL autoradiography film (Denville scientific, Metuchen, USA) was then exposed to the membrane and developed in a Kodak X-MAT 2000 processor.

### Statistical analysis

Data are expressed as mean ± SD. Differences between groups were analyzed for statistical significance with Student two-tailed, unpaired *t* test. Significance was considered achieved when the *p* value was <0.05.

## Supporting Information

Table S1 The antibodies used in this study.
(DOCX)

Table S2 The primers and probes used in this study.
(DOCX)
