# Supplementary material for: A Multifactorial Role for P. falciparum Malaria in Endemic Burkitt's Lymphoma Pathogenesis
Source: PLoS Pathog. 2014 May 29;10(5):e1004170. doi: 10.1371/journal.ppat.1004170 (PMC4038605; doi:10.1371/journal.ppat.1004170)
Supplement: Table S1 — The antibodies used in this study. (DOCX) [file ppat.1004170.s001.docx]

**Table S1. Antibodies**

Antibodies for Extracellular Staining

| Ab | Clone | Species | Source | Cat no. | Dilution  /100 µl cells | Cell number up to |
| --- | --- | --- | --- | --- | --- | --- |
| CD19APC | HIB19 (IgG1,k) | Mouse | BD Pharmingen | 555415 | 2 µl neat i.e. (1/50) | 5x10^6^ |
| CD19PE-Cy7 | HIB19 (IgG1,k) | Mouse | BD Pharmingen | 560728 | 2 µl neat i.e. (1/50) | 5x10^6^ |
| CD10PE | HI10a (IgG1,k) | Mouse | BD Pharmingen | 555375 | 2 µl neat i.e. (1/50) | 5x10^6^ |
| CD10PE-Cy5 | HI10a (IgG1,k) | Mouse | BD Pharmingen | 555376 | 10ul of (1/10) | 5x10^6^ |
| CD10APC | HI10a (IgG1,k) | Mouse | BD Pharmingen | 340923 | 2 µl neat i.e. (1/50) | 5x10^6^ |
| IgD-FITC |  | Goat | Southern Biotech | 2032-02 | 20 µl of 1/100 i.e (1/500) | 5x10^6^ |
| IgD-PE |  | Goat | Southern Biotech | 2032-09 | 2.5 µl of 1/100 i.e  (1/4,000) | 2x10^6^ |

Primary antibodies for Intracellular staining

| Ab | Clone | Species | Source | Cat no. | Dilution /100 µl cells |
| --- | --- | --- | --- | --- | --- |
| c-Myc-FITC | 9E11(IgG2ak) | Mouse | Santa Cruz | Sc-47694 | 10 µl of 1/ 100 |
| c-Myc | 9E11(IgG2ak) | Mouse | Invitrogen | AHO0052 | 10 µl of 1/ 100 |
| AID | ZA001 (IgG1k) | Mouse | Invitrogen (Zymed Labs) | [x]-18-7460 | 15 µl of (1/100) |
| Bcl6 | PG-B6P(IgG1k) | Mouse | Novus Biological | NB120-17249 | 15 µl of (1/100) |
| Bcl6 | P1F6(IgG1) | Mouse | Abcam Inc | Ab9479 | 15 µl of (1/100) |
| Isotype ctl | (IgG1) | Mouse | Caltag /Invitrogen | MG100 | 15 µl of (1/40) |
| Isotype ctl-FITC | (IgG2a) | Mouse | Santa Cruz | Sc-2856 | 10 µl of (1/25) |
| Isotype ctl | (IgG2a) | Mouse | Caltag /Invitrogen | MG2a00 | 10 µl of (1/50) |

Primary antibodies for western blot

| Antibody | Clone | Species | Source | Cat no. | Dilution /100 µl cells |
| --- | --- | --- | --- | --- | --- |
| GAPDH-HRP | 14C10 (IgG) | Rabbit | Cell signaling | 3683 | 1/5,000 in 5% milk in TBST |
| c-Myc-HRP | 9E10 | Mouse | Santa Cruz | sc-40 | 1/1,000 in milk in TBST |

c-Myc peptide (product number M2435, Sigma, St. Louis, USA) specific for mouse monoclonal anti-c-Myc 40ul/20ml Antibody solution

Secondary Antibodies of Intracellular staining

| Antibody | Species | Cat/ Pdt no. | Source | Dilution/ 100 µl cells |
| --- | --- | --- | --- | --- |
| Intracellular staining | | | | |
| Goat F(ab’)2 Anti-mouse IgG-APC | Goat | F0101B | R and D Systems, Inc | 5ul Neat |
| Goat anti-mouse IgG kappa-FITC | Goat | 1050-01 | Southern Biotech | 15 µl of 1/100 |
| Goat Polyclonal anti-mouse IgG-FITC | Goat | NB720-F | Novus Biologicals | 15 µl of 1/100 |
