# Supplementary material for: A Multifactorial Role for P. falciparum Malaria in Endemic Burkitt's Lymphoma Pathogenesis
Source: PLoS Pathog. 2014 May 29;10(5):e1004170. doi: 10.1371/journal.ppat.1004170 (PMC4038605; doi:10.1371/journal.ppat.1004170)
Supplement: Table S2 — The primers and probes used in this study. (DOCX) [file ppat.1004170.s002.docx]

**Table S2 Primers and Probes**

| Gene | Forward (For) and Reverse (Rev) | Probe |
| --- | --- | --- |
| W | For: AGTGGGCTTGTTTGTGACTTCA | TTACGTAAGCCAGACAGCAGCCAATTGTC |
|  | Rev: GGACTCCTGGCGCTCTGAT |  |
| 20x Taqman Pre-developed assay Primer-FAM Probe mix (Applied Biosystems) | | |
|  | Assay number |  |
| AID | Hs 00221068_m1 AICDA |  |
| c-Myc | Hs 00905027_m1 MYC |  |
| β-actin | Part no. 4333762T |  |

All probes are 6-FAM-TAMRA unless otherwise noted
